# A Bayesian Outbreak Detection Method for Influenza-Like Illness

**DOI:** 10.1155/2015/751738

**Published:** 2015-09-06

**Authors:** Yury E. García, J. Andrés Christen, Marcos A. Capistrán

**Affiliations:** Centro de Investigación en Matemáticas, A.C., Jalisco S/N, Colonia Valenciana, 36240 Guanajuato, GTO, Mexico

## Abstract

Epidemic outbreak detection is an important problem in public health and the development of reliable methods for outbreak detection remains an active research area. In this paper we introduce a Bayesian method to detect outbreaks of influenza-like illness from surveillance data. The rationale is that, during the early phase of the outbreak, surveillance data changes from autoregressive dynamics to a regime of exponential growth. Our method uses Bayesian model selection and Bayesian regression to identify the breakpoint. No free parameters need to be tuned. However, historical information regarding influenza-like illnesses needs to be incorporated into the model. In order to show and discuss the performance of our method we analyze synthetic, seasonal, and pandemic outbreak data.

## 1. Introduction

An important issue in public health is timely epidemic outbreak detection. Outbreak surveillance and monitoring are usually done by gathering official data reported by hospitals and clinics through medical consultation. One of the most frequent causes of medical consultation in all countries is influenza-like illness (ILI) or acute respiratory infection (ARI) [1–3]. ILI are responsible for substantial morbidity and mortality each year [3]. Seasonal influenza occurs throughout the world, and it is ranked as a leading cause of death for people below 4 and above 65 years of age and it is among the 10 top causes of death in almost all age groups [4, 5].

Early outbreak detection is necessary in order to take suitable control measures. Outbreaks correspond to breakpoints in surveillance data sets. Substantial research efforts have been devoted to this topic, inspired by a variety of statistical techniques including regression methods, time-series models, and statistical process control approaches and extensions to those fields that involve space-temporal studies and multivariate methods or techniques that include Bayesian inference [6, 7]. Comprehensive reviews of the field are presented by Unkel et al. [8], Sonesson and Bock [9], Brookmeyer and Stroup [10], and Watkins et al. [11]. Each of these papers presents a classification of methods used for outbreak detection. In general, outbreak methods use threshold values or threshold intervals to signal an alert, all based on historical data.

There are methods based on linear regression with model selection using criteria like AIC or BIC. However, outbreak detection is made under uncertainty, as noise is present in early signals of influenza surveillance [12]. Statistical methods that ignore this uncertainty may result in overconfident predictions. Bayesian methods provide a way to account for uncertainty in both data and model selection [13]. In this paper we introduce a Bayesian outbreak detection using regression models with model selection based on Bayes factors; see Hoeting et al. [13] for a review. Examples of Bayesian model comparison in linear models are [14, 15]. Smith and Spiegelhalter [16] present a review of selection criteria for linear models in terms of Bayes factors. Guo and Speckman [17] examine consistency of Bayes factors in the comparison problem for linear models. One key difference from most other methods is that the method introduced in this paper is not based on historical data alone, but rather on the exponential nature of an epidemic outbreak. For the purposes of this paper, prior information regarding influenza-like illnesses was used to build prior distributions which in turn are useful to estimate the Bayes factors for model selection.

The paper is organized as follows.  Section 2 describes the results that lead to the outbreak detection method proposed in this paper.  Section 3 applies the proposed method to synthetic and real data sets.  Section 3.3 discusses the feasibility of our approach. Finally, Section 4 summarizes our findings and offers some perspectives.

## 2. Materials and Methods

**Figure 1:**
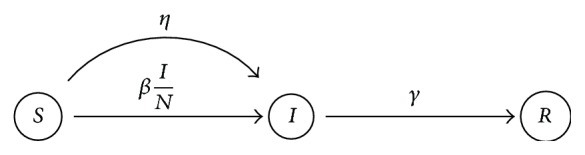
*SIR* epidemic model. Parameter *β* is the contact rate, *γ* is the recovery rate, and *η* accounts for infections due to imported cases. No births or deaths are taken into account given the time frame of an epidemic outbreak (few months for ILI).

Let us consider the epidemic process outlined in Figure 1. Let *S*(*t*), *I*(*t*), and *R*(*t*) denote the number of susceptible, infected, and recovered individuals at time *t* and the population size *N*(*t*) = *S*(*t*) + *I*(*t*) + *R*(*t*). The deterministic *SIR* model, without imported infections, that is, *η* = 0, is defined through the following ODE system [18]:

(1) dStdt=−βINS,dI(t)dt=βINS−γI,dR(t)dt=γI.

*β* is the per capita contact rate between susceptible and infected individuals and *γ* is the infection recovery rate. At the onset of an epidemic outbreak the number of infected individuals is small (relative to *N*); that is, *I*(*t*_0_) = *I*_0_ ≈ 0 and *R*(*t*_0_) = 0 at initial time *t*_0_. Therefore *S*(*t*_0_) ≈ *N* and

(2) $$\begin{array}{c} \frac{dI}{dt}=\beta \frac{I}{N}S-\gamma I\approx \left(\beta -\gamma \right)I \end{array}$$

for *t* ≈ *t*_0_; consequently

(3) $$\begin{array}{c} I\left(t\right)\approx I_{0}\exp\left(\beta -\gamma t\right)=I_{0}\exp\left(\gamma \left(R_{0}-1\right)t\right). \end{array}$$

Here *R*_0_ = *β*/*γ* is the basic reproductive number, which is defined as the expected number of secondary infections caused by an infectious individual in a totally susceptible population during the time the individual spends in the infectious compartment. An epidemic may occur if *R*_0_ is greater than one, while a basic reproductive number smaller than one will not sustain an epidemic; see [18]. Of note, the basic reproductive number does not change if *η* ≠ 0.

In the remainder of the paper we write Δ*R*_0_ = *R*_0_ − 1. Thus *I*(*t*) ≈ *I*_0_exp⁡(*γ*Δ*R*_0_ *t*) and therefore

(4) $$\begin{array}{c} \log\left(I\left(t\right)\right)\approx \log\left(I_{0}\right)+\gamma \Delta R_{0}t. \end{array}$$

That is, the logarithm of the number of infected individuals is linear in *t* during an epidemic outbreak.

On the other hand, outside epidemic outbreaks we expect that the number of infected reported cases varies around a background level, either around zero or an average number of reports as it is the case in influenza-like illnesses (ILI reports, examples to be analyzed in Section 3). By chance, the number of infected persons reports may vary around the average, with temporary runs going up (or down). In such a case we may fit a linear model in the original scale; namely,

(5) $$\begin{array}{c} I\left(t\right)\approx a+bt. \end{array}$$

The basis for our approach is to compare models (4) and (5), with a short run of reports, using the machinery of Bayesian model selection (see Section 2.1). If the exponential (i.e., linear in log scale) model is selected, it will signal the possible start of an epidemic outbreak. It will be crucial to properly code in the prior distribution for Δ*R*_0_ and *b* a clear separation between the two models, since for small values of Δ*R*_0_ both models may be quite similar (since *e* ^*x*^ ≈ 1 + *x*, for small *x*). We explain the model selection and prior selection in the following sections.

### 2.1. Bayesian Model Comparison

Given a data set of reported cases *I*(*t*_*i*_), *i* = 1,2,…, *k* at times *t*_*i*_, we consider a sliding window of *n* consecutive reports *I*(*t*_*i*_) to compare the statistical models defined by expressions (4) and (5). Before the outbreak, a linear model explains better the reported cases. On the other hand, during the early phase of the epidemic outbreak the number of infected individuals grows exponentially; thus the exponential model should be selected by the Bayes factors and the onset of the outbreak detected. Next we present an outline of Bayes factors and Bayesian model comparison and the basis for our approach.

Given two hypotheses *H*_1_ and *H*_2_ corresponding to the alternative models *M*_1_ and *M*_2_ for data *D* and parameters *θ*_1_ and *θ*_2_, the posterior distribution in each case is *f*(*θ*_*j*_∣*D*) = *p*(*θ*∣*H*_*i*_)*p*(*D*∣*θ*, *H*_*i*_)/*p*(*D*∣*H*_*i*_), *j* = 1,2. Here *p*(*θ*_*j*_∣*H*_*i*_) and *p*(*y*∣*θ*_*j*_, *H*_*i*_) are the prior and likelihood for model *i* and

(6) $$\begin{array}{c} p\left(D\;|\;H_{i}\right)=\int p\left(\theta \;|\;H_{i}\right)p\left(y\;|\;\theta ,H_{i}\right)d\theta \end{array}$$

is the normalization constant in each case. The basis of Bayesian model selection is that we can calculate the posterior distribution that each model, or each hypothesis, *H*_*i*_, is true. Namely, from Bayes's theorem we have

(7) $$\begin{array}{c} p\left(H_{i}\;|\;D\right)=\frac{p\left(D\;|\;H_{i}\right)p\left(H_{i}\right)}{p\left(D\;|\;H_{1}\right)p\left(H_{1}\right)+p\left(D\;|\;H_{2}\right)p\left(H_{2}\right)}, \end{array}$$

where *p*(*H*_*i*_) is the prior probability assigned for model *i*. The Bayes factor (*B*_1,2_) comparing these two models is given by the odds ratio of model *M*_1_ versus model *M*_2_; that is,

(8) $$\begin{array}{c} B_{1,2}=\frac{p\left(H_{1}\;|\;D\right)}{p\left(H_{2}\;|\;D\right)}=\frac{p\left(D\;|\;H_{1}\right)p\left(H_{1}\right)}{p\left(D\;|\;H_{2}\right)p\left(H_{2}\right)}. \end{array}$$

Intuitively, the Bayes factor provides a measure of whether data *D* have increased or decreased the odds on *H*_1_ versus *H*_2_. Thus *B*_1,2_ > 1 signifies that *H*_1_ (or *M*_1_) is relatively more probable than *H*_2_ (or *M*_2_) given *D* [19]. The optimal decision is therefore to choose the model with the highest posterior probability, that is, model 1 if *B*_1,2_ > 1 and model 2 otherwise.

Note that Bayes factors do not make sense when using improper priors (due to unspecified constants) and are sensitive to vague or default a priori distributions; see [20]. However, in this paper we use strong and informative (and indeed proper) priors aimed at distinguishing both models. Therefore the mentioned issues, thoroughly discussed in the Bayesian literature, should be of no concern in the current setting.

Let us denote by *M*_1_ the exponential model in (4) and *M*_2_ the linear model given in (5). Let *D* be the data at hand, either *I*(*t*_*i*_) for model 1 or log⁡*I*(*t*_*i*_) for model 2, *i* = 1,2,…, *k*. Then we assume

(9) $$\begin{array}{c} D\sim N_{n}\left(X\theta ,\sigma^{2}I_{n}\right). \end{array}$$

Thats is, *D* ∈ *ℝ* ^*n*^ follows a normal distribution with mean *Xθ* and covariance matrix *σ* ^2^ *I*_*n*_, where *I*_*n*_ is the identity matrix; *X* ∈ *ℝ* ^*n*×2^ and *θ* ∈ *ℝ* ^2^ are the design matrix and the parameter vector, respectively. We will require a different design matrix *X* and prior distributions, for each model *M*_*i*_.

To perform a standard conjugate Bayesian analysis on this linear model [19, 21, 22] we proceed as follows; please see Appendix A for more details. We use the Normal-Inverse Gamma (NIG) prior distribution:

(10) $$\begin{array}{c} \theta ,\sigma^{2}\sim \text{NIG}\left(\theta_{0},\Sigma_{0},\alpha_{0},\beta_{0}\right); \end{array}$$

*θ*_0_ corresponds to the location parameter, Σ_0_ is the covariance matrix (for  *θ*∣*σ* ^2^ ~ *N*_2_(*θ*_0_, *σ* ^2^Σ_0_)), and *α*_0_ and *β*_0_ denote the parameters of the Inverse-Gamma distribution (for *σ* ^2^ ~ InvGa(*α*_0_, *β*_0_)), in the usual way. The posterior distribution results in a NIG(*θ*_*n*_, Σ_*n*_, *α*_*n*_, *β*_*n*_), where

(11a) $$\begin{array}{c} \theta_{n}={\left(\Sigma_{0}^{-1}+X^{T}X\right)}^{-1}\left(\Sigma_{0}^{-1}\theta_{0}+X^{T}D\right), \end{array}$$

(11b) $$\begin{array}{c} \Sigma_{n}={\left(\Sigma_{0}^{-1}+X^{T}X\right)}^{-1}, \end{array}$$

(11c) $$\begin{array}{c} \alpha_{n}=\alpha_{0}+\frac{n}{2}, \end{array}$$

(11d) $$\begin{array}{c} \beta_{n}=\beta_{0}+\frac{1}{2}\left[\theta_{0}^{T}\Sigma_{0}^{-1}\theta_{0}+D^{T}D-\theta_{n}^{T}\Sigma_{n}^{-1}\theta_{n}\right]. \end{array}$$

The normalization constant in (6), required by the Bayes factor, is

(12) pD=∬pD ∣ θ,σ2pθ,σ2dθ dσ2=2π−(n+p)/2Γ(αn)2π−p/2Γ(α0)βn−αnΣnβ0−α0Σ0

(see Appendix A for more details).

From (4) and (5) it is clear that the design matrices *X* are

(13) $$\begin{array}{c} \left(\begin{array}{cc} 1 & 0\\ 1 & \gamma \\ 1 & 2\gamma \end{array}\right),\;\;\left(\begin{array}{cc} 1 & 0\\ 1 & 1\\ 1 & 2 \end{array}\right) \end{array}$$

for the log-linear (exponential) and linear models, with *θ* ^*T*^ equal to (log⁡(*I*_0_), Δ*R*_0_) and (*a*, *b*), respectively.

Other relevant parameters are explained and set in Table 1. In the following section we discuss and establish prior distributions for each model, setting the hyperparameters of the prior NIG distribution.

### 2.2. Prior Distributions

As mentioned in Section 2.1, it is crucial to separate both models through a prior distribution that distinguishes clearly the exponential growth from a linear fluctuation. The basic reproduction number *R*_0_ plays a central role in the prior information. Here, prior information of our approach is set for influenza-like illnesses; other prior specifications could be attempted for another type of epidemic outbreaks. It is known that for seasonal influenza *R*_0_ is approximately 1.5 [23]; therefore prior expectation for Δ*R*_0_ will be centered at 0.5. Moreover, in calibrating our models we have found that the bigger the population size *N* the sharper the prior needed, where the prior variance should decrease as 1/*N*. This rule is in agreement with standard hypothesis in physics; in a well mixed system the amplitude of fluctuations scales like the square root of the system size [24].

For each data window, we first subtract its corresponding mean, for either the logged or the original data, and center the prior linear model around 0. Consequently, the hyperparameters *θ*_0_ and Σ_0_ for the NIG prior are set to

(14) $$\begin{array}{c} \left(\begin{array}{c} 0\\ \frac{1}{2} \end{array}\right),\;\left(\begin{array}{cc} \log{\left(10\right)}^{2} & 0\\ 0 & \frac{1}{N} \end{array}\right),\;\left(\begin{array}{c} 0\\ 0 \end{array}\right),\;\left(\begin{array}{cc} 10^{2} & 0\\ 0 & 2 \end{array}\right), \end{array}$$

for the log-linear (exponential) and linear models, respectively. The outbreak detection method introduced here is robust to other reasonable settings for these hyperparameters. The only critical value is the variance for Δ*R*_0_, which, as mentioned above, needs to be adjusted with the population size as 1/*N*.

The remaining hyperparameters are set to *α*_0_ = (1/2)(*n* − *p*) and $\beta_{0}=\left(1/2\right)(n-p){\hat{\sigma }}^{2}$, where ${\hat{\sigma }}^{2}$ is the observed variance in the data window, for either the logged or the original data. Thus, the prior variance is centered near the observed variance for each model.

Indeed, in a pure inference scenario it is questionable to use data driven prior distributions. However, in the current setting it is desired to distinguish between the linear and exponential models and not in fact the estimation of the regression parameters themselves, which are regarded as nuisance. By subtracting the mean and centering the prior of *θ*_1_ (either to Δ*R*_0_ or to *b*) to 0 and by setting a priori $E(\sigma^{2})\approx {\hat{\sigma }}^{2}$ we are helping the inference of the regression parameters in each case (and equally for both models). This is a key feature of the proposed approach, since we will use a small window of three consecutive reports, and uncentered priors would blur the relative weight of each model, rendering the model comparison useless. Overall, the prior distribution selection at this stage should be regarded as a pragmatic approach to making the outbreak detection procedure work.

Once the outbreak is detected we may then try to estimate *R*_0_ using the data window at hand. Again, since the data set is very small, we will use a noninformative prior (see [19]) and use the marginal posterior for the regression parameters of the log-linear (exponential) model to estimate *R*_0_. The corresponding marginal posterior for the whole *θ* = (log⁡(*I*_0_), Δ*R*_0_)^*T*^ parameter is $\text{S}{\text{t}}_{p}(\hat{\theta },0.5(X^{T}X)(n-2){\hat{\beta }}_{n}^{-1},n-2)$, where ${\hat{\theta }}_{n}={\left(X^{T}X\right)}^{-1}X^{T}D$ and ${\hat{\beta }}_{n}=0.5{\left(I-X{\hat{\theta }}_{n}\right)}^{T}D$ (indeed, *D* is the logged data). The marginal distribution of any one of the entries of *θ* is a univariate Student *t* distribution. We are interested in *θ*_2_ (corresponding to Δ*R*_0_); thus $\theta_{2}\sim \text{St}{\left(({\hat{\theta }}_{n}\right)}_{2},s^{2}{\left(X^{T}X\right)}_{22},n-p)$. We will use the posterior expected value, ${\hat{\theta }}_{2}={\left({\hat{\theta }}_{n}\right)}_{2}$, of this posterior marginal to estimate *R*_0_; namely, ${\hat{R}}_{0}={\hat{\theta }}_{2}+1$. Also, since *γ* is fixed an estimator for *β* can be produced with $\hat{\beta }=({\hat{\theta }}_{2}+1)\gamma$.

In Section 3 we compute *B*_12_ over a moving window of four consecutive data points, that is, *N* = 4, to decide whether changes are due to data oscillations (linear model is selected and *B*_12_ < 1), or the onset of exponential growth occurs (the exponential model is selected and *B*_12_ > 1) and an epidemic outbreak is expected.

## 3. Results

We have tested the predictive capacity of the outbreak detection method proposed in this paper with real and synthetic data sets. The real data sets used are from the Spanish influenza outbreak in San Francisco, USA, in 1918 (see [25]) and data of the acute respiratory illnesses (ARI) from San Luis Potosí, México (see Noyola and Arteaga-Domínguez [26]).

Outbreak information and model relevant parameters like the infection rate (*β*), the basic reproductive number (*R*_0_), and the week of outbreak were estimated. In each figure, red dots indicate three consecutive points in which the exponential model is selected over the linear model; that is, *B*_12_ > 1. Grey points indicate one single four-point window in which *B*_12_ > 1. As explained in the previous section, once the outbreak is detected we use the log-linear model, with a noninformative prior, to produce estimators for both *R*_0_ and *β*.

### 3.1. Synthetic Data Analysis

**Figure 2:**
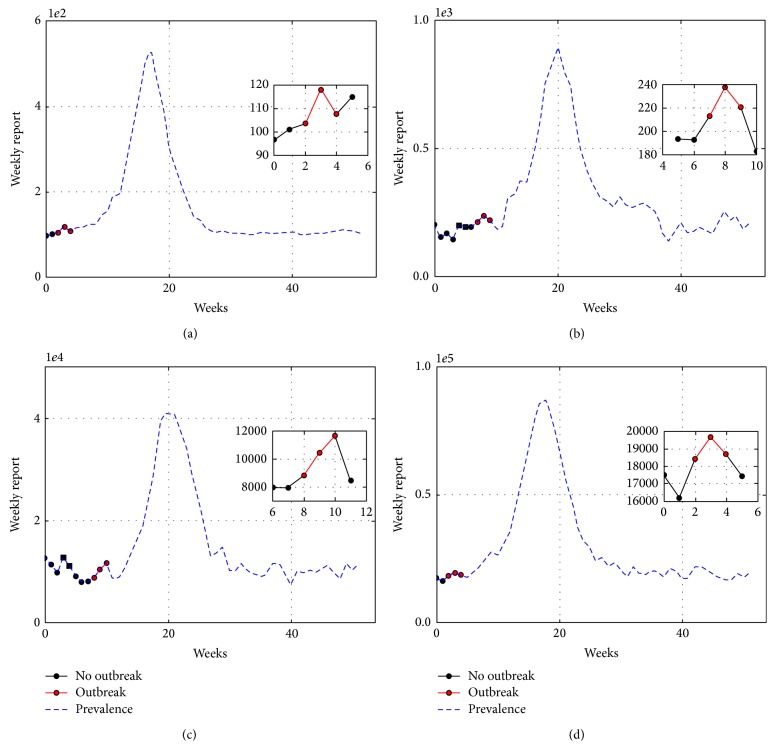
Outbreak detection for population sizes of (a) *N* = 5,000, (b) *N* = 10,000, (c) *N* = 500,000, and (d) *N* = 1,000,000. Data was generated with a realization of a *SIR* model with demographic stochasticity and imported cases. Outbreaks detection improves as the population size grows.

To create synthetic data we have avoided committing an “Inverse Crime” [27]. Synthetic data was produced with a closely related but different model to the one assumed in (4) or (5) to be producing the infectious reports. Namely, we use the Gillespie algorithm to make a realization of the *SIR* epidemic model with demographic stochasticity [28]. Initially all individuals are susceptible and the epidemic outbreak is due to imported cases. The frequency of imported cases is controlled with parameter *η*; see Figure 1. Of note, the deterministic model (1) is the mean field equation of this stochastic *SIR* model. Moreover, in a real scenario data is accumulated over the reporting time frame (daily, weekly, etc., reports for infected persons). We then accumulate the simulated data over the reported time frame to produce the synthetic infectious reports *I*(*t*_*i*_). Also, a linear autoregressive process is added to the synthetic data to simulate a background of diseases caused by other agents, as it is the case of influenza-like illness. Simulations have *R*_0_ = 1.5, *γ* = 1/7 (days); the rate of imported cases is *η* ∈ [10^−7^, 10^−4^] depending on the population size *N*. Reports are accumulated weekly. Some examples are presented in Figure 2 and the estimates for *R*_0_ and *γ* are presented in Table 2.

### 3.2. Real Data Analysis

Real surveillance data sets account for medical consultation cases. These numbers represent infected persons seeking medical attention at health centers. For influenza, it is estimated that as low as 17% of the infected population seek medical consultation and approximately 75% of people with seasonal or pandemic influenza do not exhibit symptoms [29]. However, under normal circumstances reports are proportional to the actual number of infected people and exponential growth in the number of infected people will be shown as such in the reported cases. In the following examples we do not explicitly model subreporting, obtaining good results in all cases.

**Figure 3:**
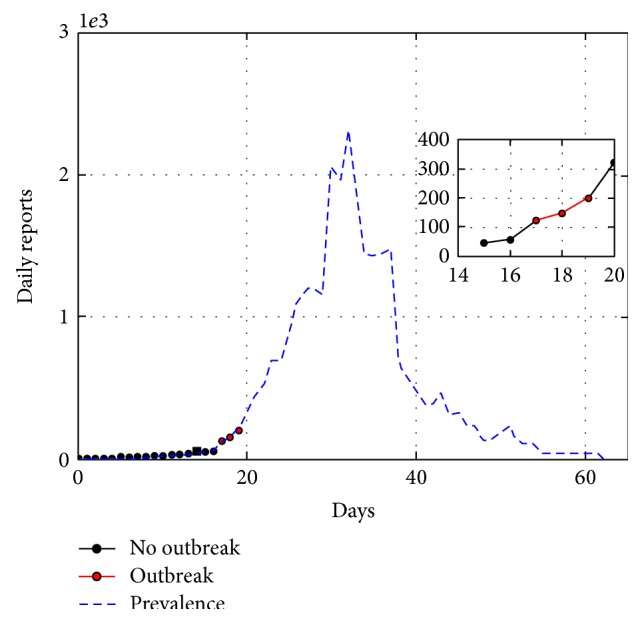
Spanish influenza in San Francisco, USA, 1918. Population 550000. Outbreak spanned from September 24th to November 24th. Method detected outbreak on the 17th day of the outbreak (October 10th). Estimated parameters are *β* = 0.53 and *R*_0_ = 3.7.

The Spanish influenza of 1917-18 was a pandemic considered among the most devastating ones in history [30, 31].  Figure 3 shows a data set corresponding to San Francisco, USA, spanning from September 24th to November 24th.

Our detection method identifies an outbreak on October 10th. The estimated parameters associated with this epidemic are $\hat{\beta }=0.53$ and ${\hat{R}}_{0}=3.7$. Both the estimated *R*_0_ and outbreak day are comparable with the values calculated by Chowell et al. [23].

**Figure 4:**
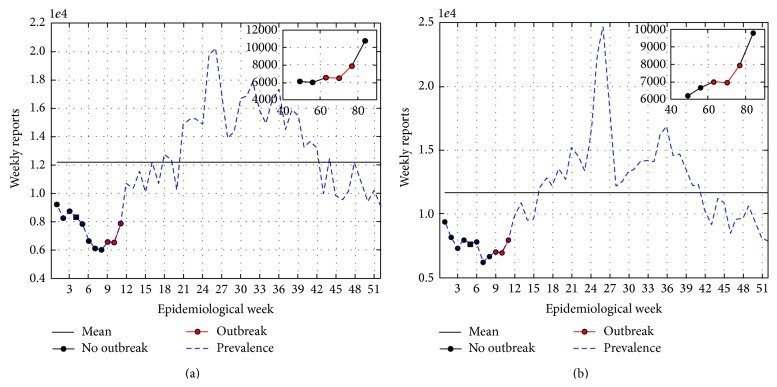
ARI reports from SLP, Mexico, winter seasons of (a) 2000-2001, $\hat{\beta }=0.22$, ${\hat{R}}_{0}=1.57$, outbreak detected at epidemiological week 8, and (b) 2003-2004, $\hat{\beta }=0.19$, ${\hat{R}}_{0}=1.37$, outbreak detected at epidemiological week 8.

Data of acute respiratory infections (ARI) in San Luis Potosí, México, are available in Noyola and Arteaga-Domínguez [26]. Here, we analyze ARI weekly reports from the winter seasons of 2000 to 2008. Reports refer to epidemiological weeks, for which week 1 is week 25 of the calendar year (i.e., mid June). Data for 2002-2003 and 2003-2004 winter seasons are plotted in Figure 4 along with outbreak detection results. In this series of data sets the seasonal outbreak is consistently detected between epidemiological weeks 13 and 15 with *R*_0_ between 1.3 and 2.5; see Table 3.

Of note, other questions from ARI surveillance may be addressed; for instance, when do the weekly reports of ARI exceed the historical mean? However, in this paper we limit ourselves to the introduction of the detection method and leave other questions of disease surveillance for future research.

### 3.3. Discussion

We have introduced an outbreak detection method based on Bayesian linear regression and Bayes factors. Our method performs correctly in real and synthetic examples. Undoubtedly a key component of this method is the structure of the prior information used to distinguish the exponential from the linear model. In the above examples we have focused on influenza-like illness (ILI) or acute respiratory infection (ARI). Consequently, the prior expectation for *R*_0_ was set equal to 1.5. We anticipate that other diseases may be modeled correctly using previous reports of the expected value of the basic reproductive number. We have learned that the prior variance for Δ*R*_0_ needs to reduce as 1/*N*, where *N* is the population size. This choice may be justified recalling that in a well mixed physical system fluctuations scale like the square root of the system size.

In the examples presented above the outbreak is detected in the presence of underreporting. The good performance of the method is explained considering the fact that the method is based on detecting a qualitative feature of the surveillance data instead of a quantitative threshold. Methods based on historical thresholds may have difficulties in detecting an outbreak happening within or below average historical report levels. Of note, our method uses historical data to calibrate prior distributions; for example, historical data is used to model how much we allow surveillance data to oscillate while in the autoregressive regime. Moreover, the method introduced in this paper allows us to estimate important parameters like infection rate (*β*) and the basic reproductive number (*R*_0_) which provide valuable information regarding outbreak behavior. The estimation of these quantities was made using a sliding window of three consecutive reports.

Bayesian outbreak detection was applied to two types of real data sets. It consistently succeeded in making an early detection and the estimated *R*_0_ and *β* values were in agreement with values reported in the literature.

A Python-Scipy implementation of our approach may be downloaded from http://www.cimat.mx/jac/software; a user friendly interphase is available at request from the authors.

## 4. Conclusions

Outbreak detection is an important problem in surveillance of infectious diseases. The development of robust methods of early outbreak detection remains an active research area.

In this paper we use Bayes factors to detect a breakpoint that characterizes the onset of an epidemic outbreak in influenza-like illness surveillance data. The breakpoint characterizes the change from an autoregressive regime to exponential behavior of reported cases at the beginning of an epidemic outbreak. The detection method was successfully used on synthetic and real data sets. The resulting algorithm is straightforwardly implemented. The mathematical methods behind the algorithm are simple but contrast with other proposed methods which are based on calculating thresholds and control charts. Of note, our approach has no free parameters to tune.

The prior distributions used arise from coding information available for influenza-like illness. It is apparent that the method may be applied to surveillance data of other infectious diseases, for example, acute diarrheal diseases, provided enough prior information about the disease of interest is available.

Certainly, it is important to detect outbreaks before they have fully developed, that is, when the number of cases is still low. Our outbreak detection method seems to be able to achieve an early detection of influenza-like illness outbreaks, when synthetic and real data are analyzed. Furthermore, it allows us to make quantitative estimations for important parameters regarding the epidemic. The estimated parameters in the data sets analyzed are in agreement with previously published values.

Some features like the optimal number of reports required to identify an outbreak, optimal number of consecutive Bayes factors required to call an outbreak, and so forth are left as subject of further research.

## Figures and Tables

**Table 1:** Model parameters summary of parameters used for both synthetic data generation and outbreak detection method.

Parameter	Value	Dimension	Description
*η*	100	Days	Infection importation rate
*γ*	7	Days	Infection recovery time
*n*	3	Reporting interval	Length of the window used to compare the models
*p*	2		Parameter index

**Table 2:** Estimates obtained for the detected outbreak.

*N*	${\hat{R}}_{0}$	Week of outbreak	$\hat{\beta }$
5000	1.23	2	0.17
10000	1.36	7	0.19
500000	1.91	8	0.27
1000000	1.35	14	0.19

**Table 3:** Parameters of acute respiratory infection records from San Luis Potosí 2000–2009. Population is approximately 2,000,000.

Year	${\hat{R}}_{0}$	Week of outbreak	$\hat{\beta }$
2000-2001	1.57	8	0.22
2001-2002	1.29	7	0.18
2002-2003	1.34	7	0.19
2003-2004	1.37	8	0.19
2004-2005	1.59	8	0.23
2005-2006	1.32	8	0.19
2006-2007	1.42	8	0.20
2007-2008	2.5	11	0.36

## Appendix

### A. Details on the Prior and Posterior Distributions and Obtaining the Normalizing Constants

Let us denote by *M*_1_ the linear model *I*(*t*) = *a* + *bt*, modeling the background data, and *M*_2_ the exponential model given by log⁡(*I*(*t*)) = log⁡(*I*_0_) + *γ*Δ*R*_0_ *t*, modeling the early outbreak. Let *D* be the data, either *I*(*t*_*i*_) for model 1 or log⁡(*I*(*t*_*i*_)) for model 2. Then, we assume

(A.1) $$\begin{array}{c} D\sim N_{n}\left(X\theta ,\sigma^{2}I_{n}\right); \end{array}$$

that is, *D* ∈ **R** ^*n*^, follows a normal distribution with mean *Xθ* and covariance matrix *σ* ^2^ *I*_*n*_, where *I*_*n*_ is the identity matrix, and *X* ∈ **R** ^*n*^ and *θ* ∈ **R** ^2^ are the design matrix and the parameter vector, respectively. The following details may also be found in [22].

#### A.1. The NIG Prior

To perform a standard conjugate Bayesian analysis on this linear model, we use the Normal-Inverse Gamma (NIG) prior distribution as follows:

(A.2) $$\begin{array}{c} \theta ,\sigma^{2}\sim \text{NIG}\left(\theta_{0},\Sigma_{0},\alpha_{0},\beta_{0}\right). \end{array}$$

This two-dimensional NIG distribution signifies that

(A.3) $$\begin{array}{c} \theta \;|\;\sigma^{2}\sim N_{2}\left(\theta_{0},\sigma^{2}\Sigma_{0}\right), \end{array}$$

where *θ*_0_ correspond to the a priori location parameter and Σ_0_ the a priori covariance matrix for *θ* and *α*_0_ and *β*_0_ denote the hyperparameters for the a priori Inverse-Gamma distribution for *σ* ^2^; consider

(A.4) $$\begin{array}{c} \sigma^{2}\sim \text{IG}\left(\alpha_{0},\beta_{0}\right). \end{array}$$

The functional form of this prior distribution is given by

(A.5) pθ,σ2  =pθ ∣ σ2pσ2=N2θ0,σ2Σ0×IGα0,β0  =β0α02πp/2Σ01/2Γ(α0)1σ2α0+p/2+1   ×exp⁡−1σ2β0+12θ−θ0TΣ0−1(θ−θ0)  ∝1σ2α0+p/2+1   ×exp⁡−1σ2β0+12θ−θ0TΣ0−1θ−θ0,

where Γ(·) represents the Gamma function and the IG(*α*_0_, *β*_0_) prior density for *σ* ^2^ is given by

(A.6) pσ2=β0α0Γα01σ2α0+1exp⁡−β0σ2,σ2>0, β0>0, α0>0.

#### A.2. The Likelihood

The likelihood function for each model is defined as the joint probability of observing the data viewed as a function of the parameters; consequently

(A.7) PD ∣ θ,σ2 =NXθ,σ2In =12πσ2n/2×exp⁡−12σ2D−XθT(D−Xθ)

viewed as a function of *θ* and *σ* ^2^ and fixing *D*.

#### A.3. The Posterior NIG Distribution

The posterior distribution is defined as *p*(*θ*, *σ* ^2^∣*D*) = *p*(*θ*, *σ* ^2^)*p*(*D*∣*θ*, *σ* ^2^)/*p*(*D*), where *p*(*D*) = ∫*p*(*θ*, *σ* ^2^)*p*(*D*∣*θ*, *σ* ^2^)*dθ* *dσ* ^2^ is the marginal distribution of the data.

We have that

(A.8) pθ,σ2 ∣ D  =NIGθ0,Σ0,α0,β0×NXθ,σ2InpD  ∝β0α02πp/2Σ01/2Γ(α0)1σ2α0+p/2+112πσ2n/2   ×exp⁡−1σ2β0+12θ−θ0TΣ0−1(θ−θ0)   −12σ2D−XθTD−Xθ  ∝1σ2α0+(p+n)/2+1   ·exp⁡⁡−1σ2β0+12θ−θ0TΣ0−1θ−θ0WWWWWWIW−1σ2+D−XθT(D−Xθ).

Using the identity

(A.9) $$\begin{array}{c} u^{T}Au-2\alpha^{T}u={\left(u-A^{-1}\alpha \right)}^{T}A\left(u-A^{-1}\alpha \right)-\alpha^{T}A^{-1}\alpha \end{array}$$

we may write

(A.10) 1σ2β0+12θ−θ0TΣ0−1(θ−θ0)+D−XθT(D−Xθ)  =1σ2βn+12θ−θn∗TΣn−1θ−θn∗,

where

(A.11) $$\begin{array}{c} \theta_{n}={\left(\Sigma_{0}^{-1}+X^{T}X\right)}^{-1}\left(\Sigma_{0}^{-1}\theta_{0}+X^{T}D\right),\\ \Sigma_{n}={\left(\Sigma_{0}^{-1}+X^{T}X\right)}^{-1},\\ \alpha_{n}=\alpha_{0}+\frac{n}{2},\\ \beta_{n}=\beta_{0}+\frac{1}{2}\left[\theta_{0}^{T}\Sigma_{0}^{-1}\theta_{0}+D^{T}D-\theta_{n}^{T}\Sigma_{n}^{-1}\theta_{n}\right]. \end{array}$$

Therefore,

(A.12) pθ,σ2 ∣ D  =1σ2α0+n+p/2+1WWW×exp⁡−1σ2βn+12θ−θnTΣn−1θ−θn, pθ,σ2 ∣ D∝NIGθn,Σn,αn,βn.

#### A.4. The Normalization Constant

This is the constant required by the Bayes factor. We need to compute the distribution *p*(*D*∣*σ* ^2^) by integrating out *β* and subsequently integrate out *σ* ^2^ to obtain *p*(*D*). Accordingly,

(A.13) pD ∣ σ2=∫pD ∣ θ,σ2pθ ∣ σ2dθ=∫NXθ,σ2In×Nβ0,σ2Σ0dθ=12πσ2n+p/2Σ01/2 ·∫exp⁡⁡−12σ2D−XθT(D−Xθ)WWWWWWWi12σ2+θ−θ0TΣ0−1(θ−θ0)dθ=12πσ2n+p/2Σ01/2 ×∫exp⁡⁡−12σ2D−Xθ0TI+XΣ0XT−1(D−Xθ0)WWWWW12σ2+θ−θnTΣn−1(θ−θn)dθ=12πσ2n+p/2Σ01/2 ·exp⁡−12σ2D−Xθ0TI+XΣ0XT−1(D−Xθ0) ×∫exp⁡−12σ2θ−θnTΣn−1θ−θndθ=12πσ2n/2ΣnΣ01/2 ·exp⁡−12σ2D−Xθ0TI+XΣ0XT−1(D−Xθ0)=12πσ2n/2I+XΣ0XT1/2 ·exp⁡−12σ2D−Xθ0TI+XΣ0XT−1(D−Xθ0)=NXθ0,σ2I+XΣ0XT.

Here the matrix identity |*A* + *BDC*| = |*A*||*D*||*D* ^−1^ + *CA* ^−1^ *B*| was applied to obtain

(A.14) $$\begin{array}{c} \left|I_{n}+X\Sigma_{0}X^{T}\right|=\left|\Sigma_{0}\right|\left|\Sigma_{0}^{-1}+X^{T}X\right|=\left(\frac{\left|\Sigma_{0}\right|}{\left|\Sigma_{n}\right|}\right). \end{array}$$

Now, the marginal distribution of *p*(*D*) is obtained as follows:

(A.15) pD=∫pD ∣ θ,σ2pθ,σ2dθ dσ2=∫N(Xθ,σ2In)×NIGθ0,Σ0,α0,β0dθ dσ2=MVSt2α0Xθ,β0α0I+XΣ0XT.

In more detail, we have

(A.16) pD=∬pD ∣ θ,σ2pθ,σ2dθ dσ2=∬ND ∣ Xθ,σ2In×NIGθ,σ2 ∣ θ0,Σ0,α0,β0dθ dσ2=β0α02πp/2Σ01/2Γ(α0) ·∬1σ2αn+p/2+1  ·exp⁡−1σ2βn+12θ−θntΣn−1(θ−θn)dθ dσ2=β0α0Γ(α0)2π(n+p)/2Σ0Γ(αn)2πp/2Σnβnαn=2π−(n+p)/2Γ(αn)2π−p/2Γ(α0)βn−αnΣnβ0−α0Σ0.

Thus, the posterior distribution is

(A.17) pθ,σ2 ∣ D=p(θ,σ2)×pD ∣ β,σ2p(D)=NIGθ0,Σ0,α0,β0×NXθ,σ2IMVSt2α0Xθ,β0/α0I+XΣ0XT,

which indeed reduces (after some algebraic manipulation) to the NIG(*θ*_*n*_, Σ_*n*_, *α*_*n*_, *β*_*n*_) density.

The marginal distribution of any one of the entries of *θ*_*n*_ is a univariate Student *t* distribution. This is used and the correct parameters are described in Section 2.2 to estimate *R*_0_ and infection rate (*β*).
